# The Red Alga *Jania rubens* DM Extract Modulates Apoptotic and Inflammatory Pathways to Enhance Chemotherapeutic Response in Colorectal Cancer Cells

**DOI:** 10.3390/cimb48080759

**Published:** 2026-07-26

**Authors:** Zeina Radwan, Maysam Moussa, Shaza Khatib Ibrahim, Shaymaa Al Sharif, Rayan Kassir, Fatima El-Mched, Lara Haddad, Nadine Darwiche, Marwan El-Sabban, Hiba Mawlawi, Zeina Dassouki

**Affiliations:** 1 Department of Anatomy, Cell Biology, and Physiological Sciences, Faculty of Medicine, American University of Beirut, Beirut 1107-2020, Lebanon; zar04@mail.aub.edu (Z.R.); me00@aub.edu.lb (M.E.-S.); 2 Department of Medical Laboratory Sciences, Faculty of Health Sciences, University of Balamand, Al-Koura, Tripoli P.O. Box 100, Lebanon; maysam.moussa@balamand.edu.lb (M.M.); shazakhatib1227@gmail.com (S.K.I.); shaymaa.alsharif@std.balamand.edu.lb (S.A.S.); lara.haddad@balamand.edu.lb (L.H.); 3 Laboratory of Applied Biotechnology, AZM Center for Research in Biotechnology and Its Applications, Doctoral School for Sciences and Technology, Lebanese University, Tripoli 1300, Lebanon; rayan.alkassir@ul.edu.lb; 4 Department of Biological Science, Faculty of Science, Beirut Arab University, Tripoli P.O. Box 11-5020, Lebanon; fatima.mashad@bau.edu.lb; 5 Department of Biochemistry and Molecular Genetics, Faculty of Medicine, American University of Beirut, Beirut P.O. Box 110236, Lebanon; nd03@aub.edu.lb; 6 Faculty of Public Health III, Lebanese University, Tripoli 1300, Lebanon

**Keywords:** *Jania rubens*, red algae extract, colorectal cancer, capecitabine, irinotecan, apoptosis

## Abstract

Colorectal cancer (CRC) treatment with standard chemotherapeutics such as capecitabine (CAP) and irinotecan (IRT) is frequently limited by toxicity and resistance. To identify novel adjuvant strategies, we investigated the dichloromethane–methanol (DM) Soxhlet extract of the red alga *Jania rubens*, previously shown to exert intrinsic antiproliferative effects via reactive oxygen species (ROS) induction, inhibition of epithelial–mesenchymal transition (EMT), and suppression of TET enzymes. The present study evaluated the effect of the DM extract in combination with chemotherapeutic agents in HCT-116, Caco-2, and HT-29 colorectal cancer cell lines, assessing its potential to enhance treatment response. Co-treatment with the DM extract significantly enhanced the cytotoxic and anti-migratory effects of CAP and IRT in HCT-116 and Caco-2 cells. Combination treatment also impaired long-term clonogenic survival, suggesting the inhibition of therapy-resistant subpopulations. Flow cytometric analysis (Annexin V-FITC/PI) revealed a dose-dependent increase in apoptosis in HCT-116 cells following DM treatment. Western blot analysis further supported the pro-apoptotic activity of the DM extract by demonstrating reduced BCL-2 protein expression. The extract additionally modulated the mRNA expression levels of cytokines (TNF-α, IL-6, and IL-10), suggesting potential immunomodulatory effects in colorectal cancer cells. Notably, co-administration of IRT and the DM extract enhanced apoptosis, primarily through the downregulation of the anti-apoptotic gene BCL-2. Together, these findings indicate that the *Jania rubens* DM extract modulates multiple cellular pathways and enhances the response to conventional CRC chemotherapeutic agents.

## 1. Introduction

Colorectal cancer (CRC) is one of the most prevalent malignancies worldwide, with approximately 1,000,000 new cases reported annually [1,2]. It is among the leading causes of cancer-related mortality and one of the most commonly diagnosed cancers in both men and women, with a slightly higher incidence in men [3]. Surgery, chemotherapy, radiation, and immunotherapy are among the conventional treatments for colon cancer progression and metastasis. These treatments can be used alone or in combination, depending on the patient’s condition and disease stage [4,5]. Chemotherapy has greatly contributed to the long-term decline in mortality from CRC [6]. Conventional chemotherapeutic agents suppress tumor growth and invasion but can also adversely alter the tumor microenvironment and cause significant toxicity due to high dosing requirements. Furthermore, the survival rates of patients continue to be low, and certain individuals may experience restricted effectiveness and develop resistance to medications [4,5,6].

This is illustrated by Capecitabine (CAP), a prodrug of 5-FU. It was initially developed to improve safety, tolerability, and tumoral drug concentrations [5]. It exerts its anti-tumorigenic effect by disrupting DNA and RNA synthesis. However, CAP still leads to several serious side effects, such as cardiotoxicity, bone marrow depression, and other adverse events. [7]. Similarly, CPT-11, also known as irinotecan (IRT), has also been used to treat colorectal, lung, and brain tumors [8]. IRT exerts its anticancer effects by inhibiting topoisomerase I, leading to DNA damage during replication and increased oxidative stress [8]. The dose-limiting side effects of this drug are hematological, intestinal, and systemic toxicities, as well as severe myelosuppression [9,10]. In light of these examples, alternative treatments are currently being studied to decrease the adverse effects of conventional chemotherapy while ensuring cytotoxicity to cancer cells and anti-invasive effects.

Additionally, chemoresistance in colorectal cancer (CRC), which remains a major obstacle to successful treatment, may arise through altered drug transport, metabolic reprogramming, and the activation of pro-survival signaling pathways [11]. It is a complex, multifaceted phenomenon frequently associated with the dysregulation of key survival pathways that enable tumor cells to evade apoptosis and continue proliferating despite cytotoxic therapy.

Moreover, cancer cells often disrupt the balance between pro-apoptotic and anti-apoptotic molecules. Overexpression of anti-apoptotic BCL-2 family proteins, particularly BCL-2, contributes to chemoresistance by inhibiting mitochondrial apoptosis and allowing tumor cells to survive chemotherapy-induced damage [12,13]. These molecular alterations reduce therapeutic efficacy and contribute to disease progression and recurrence. Consequently, strategies aimed at restoring apoptotic sensitivity and suppressing survival signaling may enhance CRC cell responsiveness to conventional chemotherapeutic agents.

Alongside the abovementioned factors, inflammatory mediators have been increasingly recognized as important modulators of chemotherapeutic response in colorectal cancer [14,15]. In fact, tumor cells secrete a variety of cytokines that function in both autocrine and paracrine manners to drive tumor progression, survival, and resistance to therapy [16]. Pro-inflammatory cytokines such as IL-6 and TNF-α are known to interact with intracellular signaling cascades, including the PI3K/AKT and MAPK/ERK pathways, thereby influencing apoptotic sensitivity and treatment outcomes [17,18]. IL-6 has been associated with enhanced survival signaling and reduced responsiveness to chemotherapy, whereas TNF-α may exert context-dependent pro-apoptotic or pro-survival effects [19,20]. Moreover, IL-10 has been implicated in modulating inflammatory signaling and tumor cell adaptation to stress conditions [21]. Therefore, the modulation of cytokine expression within tumor cells may represent an additional mechanism through which chemosensitivity can be altered.

The Lebanese coast is abundant in seaweeds, where over 94 macroalgal species (green, brown, and red algae) are found [22,23]. *Jania rubens* (*J. rubens*) is the most prevalent red seaweed in the eastern Mediterranean region. We have previously demonstrated that *J. rubens’* organic extract (dichloromethane-methanol; DM Soxhlet extract) has antiproliferative and anti-migratory effects on colon cancer cells [22]. We also showed that the extract exhibits extensive anticancer properties in HCT-116 colorectal cancer cells by inducing cell cycle arrest, inhibiting the epithelial–mesenchymal transition (EMT) markers (N-cadherin and TWIST), suppressing MMP-2 and MMP-9 activity, downregulating TET (ten-eleven translocation enzyme) expression and functional outputs, and enhancing ROS (reactive oxygen species) production [24]. Furthermore, numerous bioactive substances, including flavonoids and pyrazole derivatives, were identified by GC-MS analysis and may contribute to the observed biological reactions [24].

Given its previously demonstrated multi-targeted anticancer properties, we hypothesized that the DM Soxhlet extract could act as an effective adjuvant to enhance the therapeutic efficacy of the conventional chemotherapeutics CAP and IRT. Accordingly, this study aimed to investigate its effects in combination with chemotherapeutic agents and to elucidate the underlying molecular mechanisms.

## 2. Materials and Methods

### 2.1. Preparation of the Extract

Samples of *J. rubens* were gathered at a depth of two to three meters from the Mediterranean coast of North Lebanon. After they were carefully cleaned and allowed to air-dry at room temperature, fresh seaweed was ground into a fine powder. A *J. rubens*’ herbarium voucher (AZM-1105) was kept at the Lebanese University’s Doctoral School of Science and Technology.

The DM Soxhlet extract was made by extracting the powdered algae with dichloromethane/methanol (DM, 1:1) at a high temperature for six cycles in a Soxhlet extractor. A rotary evaporator was then used to concentrate the extract. DMSO was used to dilute the resulting solid extract to a concentration of 100 mg/mL. Dulbecco’s Modified Eagle Medium (DMEM, high glucose; Sigma, St. Louis, MO, USA) or Roswell Park Memorial Institute Medium (RPMI 1640, Corning, NY, USA) was used to generate an intermediate dilution of 1000 µg/mL. This was followed by further dilutions to reach the final 100, 250 and 500 µg/mL concentrations. The concentrations of *J. rubens* DM extract used in this study were selected based on our previous dose–response analyses, where 500 µg/mL was identified as the IC_50_ value in HCT-116 cells. Lower concentrations (100 and 250 µg/mL) were included to assess dose-dependent effects and to evaluate sub-cytotoxic responses across different experimental assays [22].

To ensure that the observed effects were primarily due to the extract and not the solvent, the DMSO concentration was kept below 1% and a DMSO-only control was included. At the concentrations used, no discernible cytotoxicity was seen in wells treated with DMSO.

### 2.2. Extract Standardization and Compositional Consistency

To ensure reproducibility and minimize batch-to-batch variability, all *J. rubens* dichloromethane–methanol (DM) extract samples were prepared using a controlled and reproducible Soxhlet extraction protocol. Identical extraction parameters were applied for all batches, including solvent composition and ratio, extraction duration, temperature, and algae-to-solvent ratio. The same laboratory-grade equipment and analytical-grade solvents were used throughout the study.

Algal material was standardized at the source to reduce environmental and seasonal variability. *J. rubens* specimens were collected from the same geographical location (Qalamoun, North coastal Lebanon), at a uniform depth of 2–3 m, and during the same harvesting period (October). All samples were processed within one week of collection, thoroughly washed, shade-dried under controlled conditions, and stored in airtight containers to limit degradation prior to extraction.

To characterize the extract composition and support reproducibility, GC–MS analysis was performed on representative batches of the DM extract, as previously reported [24]. The analysis revealed a consistent profile of secondary metabolites dominated by flavonoid derivatives, including 4′-methoxyflavone, 7-methoxyflavone, and 8-methoxyflavone, and related benzopyranone structures, along with pyrazole-based and heterocyclic nitrogen-containing compounds. The relative abundance of compounds was estimated based on peak area normalization, with major constituents exhibiting the highest peak areas within the chromatogram. Compound identification was achieved by comparison with NIST library spectra, and match quality scores (“Qual”) were used to assess identification reliability. Retention times of detected compounds ranged from approximately 27 to 52 min. Importantly, similar chromatographic profiles were observed across extract batches, supporting the reproducibility of the extraction procedure.

It should be noted that GC–MS analysis provides tentative identification and semi-quantitative estimation and does not constitute full structural confirmation or standardization of individual bioactive compounds. Therefore, the observed biological effects are likely attributable to the combined activity of multiple constituents rather than a single defined compound.

### 2.3. Chemotherapies Preparation

Capecitabine (CAP, HY-B0016; MedChemExpress, Monmouth Junction, NJ, USA) and Irinotecan (IRT, HY-16562A; MedChemExpress, Monmouth Junction, NJ, USA) were prepared according to the manufacturers’ recommendations by dissolving in DMSO to stock concentrations of 1 M and 10 mM, respectively, and stored at −80 °C. Prior to treatment, the stock solutions were diluted to final working concentrations of 5 mM for CAP and 100 µM for IRT using the appropriate culture medium (DMEM, high glucose or RPMI-1640). These concentrations were selected based on preliminary dose–response analyses (MTT assay) and correspond to values near the IC_50_ in the tested colorectal cancer cell lines. The selected doses enabled the assessment of cytotoxic and combinatorial effects without inducing complete cell loss, particularly in long-term assays such as clonogenic survival.

### 2.4. Cell Lines and Cell Culture

Colorectal cancer cell lines (HCT-116, Caco-2 and HT-29) were obtained from the American Type Culture Collection (ATCC). HCT-116 and Caco-2 cells were grown in Dulbecco’s Modified Eagles Medium (DMEM, high glucose; Sigma, St Louis, MO, USA) whereas HT-29 were cultured in RPMI-1640 (Corning, NY, USA). Cells were kept in a humidified incubator at 37 °C, 5% CO_2_, and 95% air. The media were enriched with 10% fetal bovine serum and 1% penicillin–streptomycin (100 U/mL).

HCT-116 cells were selected for mechanistic assays (flow cytometry and RT-qPCR) due to their reproducible response to treatment and well-established molecular characteristics, making them a suitable model for evaluating apoptotic and signaling-related changes.

### 2.5. Cell Viability Assay

The ability of metabolically active cells to transform tetrazolium salt into formazan blue was measured by the cell viability assay, an MTT-based technique. In a 96-well plate, cells were seeded (30,000/cm^2^) and allowed to adhere until they reached 70% confluency. Following a 24 h treatment, the cells were incubated for four hours at 37 °C in the dark with MTT [3-(4, 5-dimethylthiazol-2-yl)-2, 5-diphenyltetrazolium bromide] (Sigma, St. Louis, MO, USA). Before the absorbance was measured, DMSO was applied for 15 min. Absorbances at 570 nm were measured by the ELISA microplate reader (BioTek Instruments, Winooski, VT, USA).

### 2.6. Wound Healing Assay

Wound-healing assays were performed using HCT-116 and Caco-2 colorectal cancer cells cultured in 24-well plates. *A* linear scratch was created in the cell monolayer using a sterile 200 µL pipette tip. Cells were washed twice with phosphate-buffered saline to remove debris and then treated with 250 µg/mL DM extract, 5 mM capecitabine (CAP), 100 µM irinotecan (IRT), or their respective combinations. Images of the wound area were captured at 0 h and 24 h post-treatment using a digital camera attached to an optical microscope. Wound closure was quantified using ImageJ software (version 1.54d, National Institutes of Health, Bethesda, MD, USA) and expressed as relative fold change compared to untreated control cells, which were set to 1. Experiments were performed in independent biological replicates. Relative wound closure was calculated as: $(A_{0}-A_{24})/A_{0}$, normalized to untreated control cells set to 1.

### 2.7. Crystal Violet Assay

After being seeded in 24-well plates at a density of 5000 cells per well and allowed to adhere for 48 h [25], HCT-116 or Caco-2 cells were treated for 24 h with 250 µg/mL DM, 5 mM CAP, 100 µM IRT, or their combinations. Following treatment, the cells were allowed to grow for one week to form colonies. Finally, the cells were stained with 0.1% crystal violet solution (Sigma-Aldrich, St. Louis, MO, USA; Cat. No. 94448) prepared in PBS. Colonies were defined as discrete clusters containing approximately ≥50 cells. Quantification was performed using ImageJ, applying a consistent size threshold across all conditions to exclude small cell aggregates and background staining.

### 2.8. Annexin V-FITC/Propidium Iodide (PI) Staining

Apoptosis was evaluated using Annexin V-FITC/propidium iodide (PI) staining following the manufacturer’s two-step protocol (Elabscience Biotechnology Inc., Houston, TX, USA, Cat. No: E-CK-A211). Briefly, HCT-116 cells were treated with DM 250 and 500 µg/mL or left untreated for four hours. Cells were collected and centrifuged at 300× *g* for 5 min. The supernatant was discarded, and cells were washed once with PBS, gently resuspended, and counted. Subsequently, 2.5 × 10^5^ cells were aliquoted into individual tubes and centrifuged again at 300× *g* for 5 min. After removal of the supernatant, cells were washed with PBS, centrifuged, and resuspended in 100 μL of 1× Annexin V binding buffer. Cells were then stained with 2.5 μL Annexin V-FITC and 2.5 μL PI per tube. Samples were incubated for 20 min at room temperature in the dark. Finally, 400 μL of 1× Annexin V binding buffer was added to each tube prior to flow cytometric analysis.

### 2.9. Reverse Transcription, qRTPCR, and RNA Extraction

TRIzol Reagent was used to extract total RNA, which was then separated by chloroform-induced phase separation and centrifuged for 15 min at 4 °C at 12,000× *g*. After collecting and precipitating the RNA-containing supernatant with isopropanol, the samples were centrifuged (10 min at 4 °C at 12,000× *g*). After two rounds of washing with 75% ethanol at 7500× *g*, the resultant RNA pellet was allowed to air-dry for 20 min at room temperature. After that, the pellet was mixed with 20 μL of nuclease-free water. Lastly, the samples were kept at −20 °C and RNA was measured using a Nanodrop spectrophotometer (ND-1000 Spectrophotometer, Thermo Fisher Scientific, Wilmington, NC, USA).

Using Bio-Rad Laboratories’ Iscript cDNA Synthesis Kit, 1 μg of RNA was reverse-transcribed to cDNA.

Then, real-time quantitative PCR was carried out using the SsoAdvanced™ Universal Sybr^®^ Green SuperMix (Bio-Rad) and the iTaq™ Universal Sybr^®^ Green Supermix (Bio-Rad) following the manufacturers’ protocol. The primers of the genes of interest used are:

Bad (F: 5′-ACTGAGGTCCTGAGCCGACA-3′, R: 5′-TCTGGGCTGTGAGGACAAGAT-3′), BCL-2 (F: 5′-GAACTGGGGGAGGATTGTGG-3′, R: 5′-CCGTACAGTTCCACAAAGGC-3′), GAPDH (F: 5′-GCACCACCAACTGCTTAGCA-3′, R: 5′-CTTCCACGATACCAAAGTTGTCAT-3′), as well as the housekeeping gene GAPDH.

### 2.10. Western Blot Analysis

Cells were lysed using a protein extraction buffer supplemented with protease and phosphatase inhibitor cocktails. Total protein concentration was determined using the Bradford protein assay according to the manufacturer’s instructions. Equal amounts of protein (70 µg) were mixed with 1× loading buffer and separated by SDS–PAGE using 10% resolving gels with a 4% stacking gel. Electrophoresis was performed at 90 V through the stacking gel and 120 V through the resolving gel. Proteins were transferred onto a 0.45 µm PVDF membrane at 100 V for 90 min on ice. Membranes were blocked with 5% bovine serum albumin (BSA) in Tris-buffered saline containing 0.1% Tween-20 (TBST) for 1 h at room temperature and incubated overnight at 4 °C with the appropriate primary antibodies diluted in blocking buffer (BCL-2; Santa Cruz Biotechnology; SC 783). After three washes with TBST (10 min each), membranes were incubated with an HRP-conjugated anti-rabbit IgG secondary antibody (Cell Signaling Technology, Danvers, MA, USA; Cat. No. 7074S) for 1 h at room temperature. Membranes were washed five additional times with TBST, and protein bands were visualized using an enhanced chemiluminescence (ECL) substrate and detected using a ChemiDoc imaging system (Bio-Rad, Hercules, CA, USA). Band intensities were quantified by densitometric analysis using ImageJ software and normalized to β-actin.

### 2.11. Statistical Analysis

All statistical analyses were performed using GraphPad Prism 10 (version 10.0; GraphPad Software, Boston, MA, USA). Quantitative data are presented as mean ± SD from at least three independent biological replicates, unless otherwise stated. Statistical significance was defined as * *p* < 0.05, ** *p* < 0.01, *** *p* < 0.001, and **** *p* < 0.0001.

## 3. Results

### 3.1. DM Soxhlet Extract Potentiates CAP-Induced Cytotoxicity in CRC Cells

**Figure 1:**
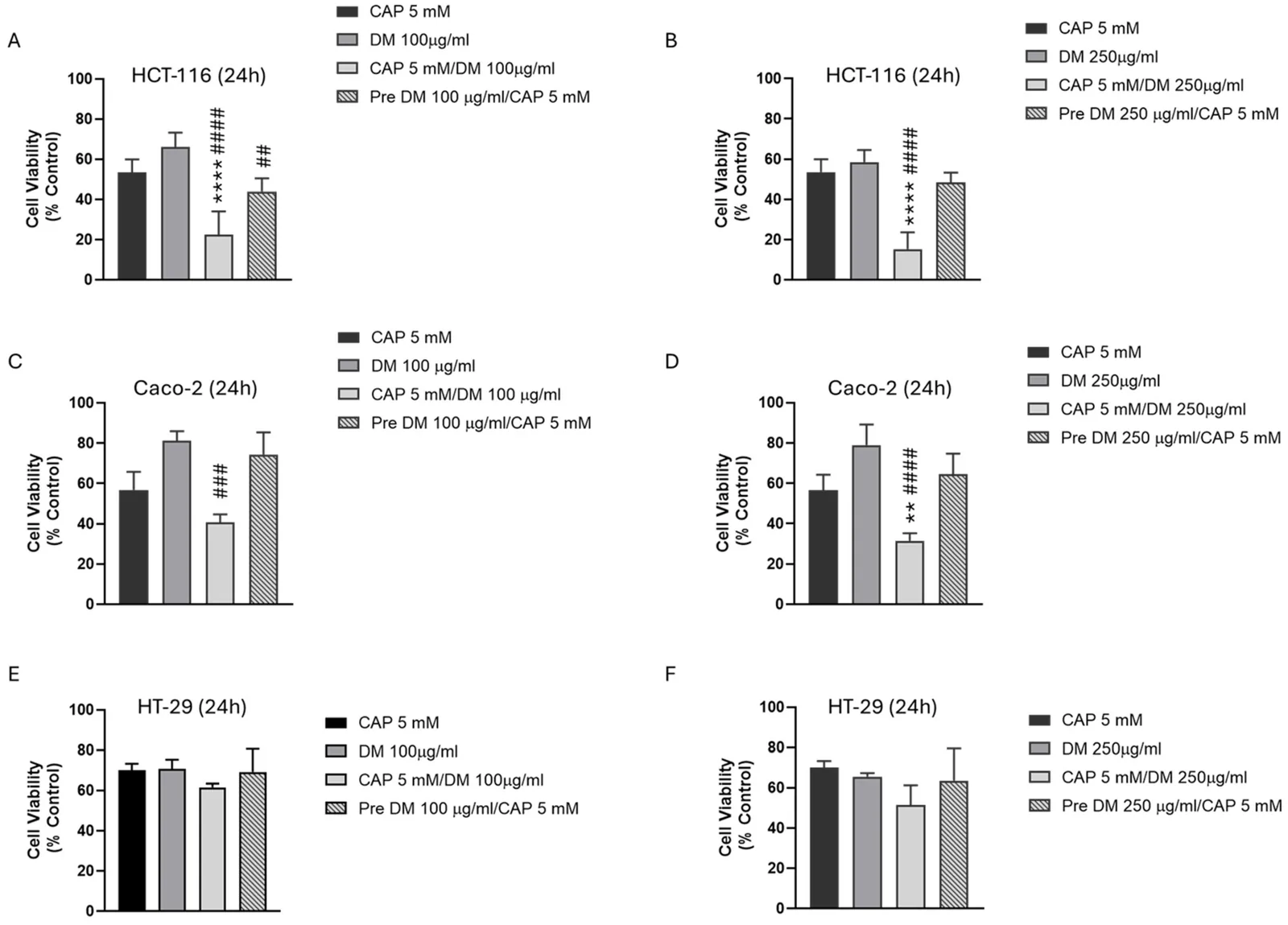
DM Soxhlet extract potentiates CAP-induced cytotoxicity in CRC cells. MTT assay was performed to assess the viability of HCT-116, Caco-2, and HT-29 cells following 24 h of treatment under various conditions. Cells were treated with CAP (5 mM), the DM extract (100 or 250 μg/mL), or their combinations for 24 h, or pretreated with 100 μg/mL or 250 μg/mL DM extract for 3 h prior to CAP (5 mM) exposure for 24 h. (**A**,**B**) HCT-116 cells; (**C**,**D**) Caco-2 cells; (**E**,**F**) HT-29 cells. Cell viability was expressed as a percentage relative to the untreated control (set at 100%). Data are presented as mean ± SD (n ≥ 3 independent experiments). ** *p* < 0.01, **** *p* < 0.0001 vs. CAP-treated group; ## *p* < 0.01, ### *p* < 0.001, #### *p* < 0.0001 vs. DM-treated group. DM: Dichloromethane/Methanol; CAP: Capecitabine.

To investigate the potential of the DM Soxhlet extract as an adjuvant in CRC treatment, HCT-116, Caco-2, and HT-29 cells were treated with CAP (5 mM), the DM extract (100 or 250 μg/mL), or their combinations for 24 h. The combined treatments resulted in significantly greater cytotoxic effects than individual treatments in HCT-116 and Caco-2 cells. However, in HT-29, no significant changes were observed with the combined treatment (Figure 1E,F). The DM extract reduced viability to 66% (100 μg/mL) and 60% (250 μg/mL) in HCT-116 cells. When co-administered with 5 mM CAP, cell viability declined to 22% and 15%, respectively (Figure 1A,B). Similarly, combining 5 mM CAP with 250 μg/mL DM extract decreased viability in Caco-2 cells from 60% to 45% (Figure 1D).

We also explored whether pretreating cells with the DM extract could enhance CAP efficacy. Cells were pre-incubated with 100 or 250 μg/mL DM extract for 3 h, after which the extract was removed and replaced with 5 mM CAP for 24 h. Notably, pretreatment with DM extract before CAP administration did not enhance cytotoxicity compared to CAP alone in any of the tested cell lines (Figure 1).

### 3.2. Combined Treatment with DM Soxhlet Extract and IRT Increases Cytotoxicity in CRC Cells

**Figure 2:**
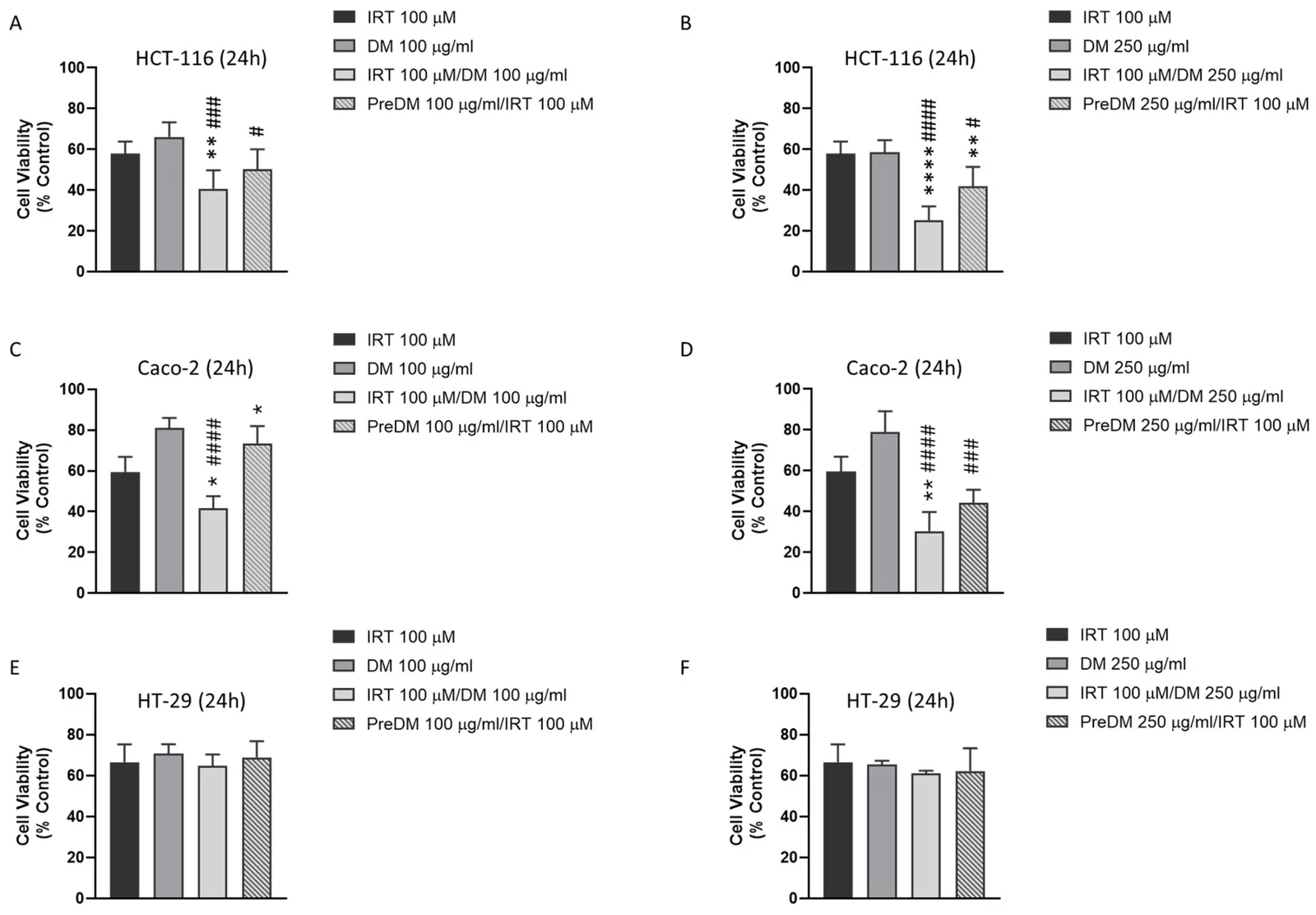
Combined treatment with DM Soxhlet extract and IRT increases cytotoxicity in CRC cells. MTT assay was performed to assess the viability of HCT-116, Caco-2, and HT-29 cells following 24 h treatment under various conditions. Cells were treated with IRT (100 µM), the DM extract (100 or 250 μg/mL), or their combinations for 24 h, or pretreated with 100 μg/mL or 250 μg/mL DM extract for 3 h prior to IRT (100 µM) exposure for 24 h. (**A**,**B**) HCT-116 cells; (**C**,**D**) Caco-2 cells; (**E**,**F**) HT-29 cells. Cell viability was expressed as a percentage relative to the un-treated control (set at 100%). Data are presented as mean ± SD (n ≥ 3 independent experiments). * *p* < 0.05, ** *p* < 0.01, **** *p* < 0.0001 vs. CAP-treated group; # *p* < 0.05, ### *p* < 0.001, #### *p* < 0.0001 vs. DM-treated group. DM: Dichloromethane/Methanol; IRT: Irinotecan.

We also examined the effect of DM Soxhlet in combination with IRT. HCT-116, Caco-2, and HT-29 cells were exposed to IRT (100 µM), DM extract (100 or 250 μg/mL), or the combination of both for 24 h. The results revealed that the combination treatments were significantly more inhibitory of cell viability in both HCT-116 and Caco-2 cells. HT-29 cells, however, showed less improvement with the combination (Figure 2E,F).

In HCT-116 cells, treatment with 100 µM IRT decreased viability to 58%. But co-treatment with 100 μg/mL or 250 μg/mL DM extract decreased viability further to 41% and 25%, respectively (Figure 2A,B). Similarly, in Caco-2 cells, co-treatment with 100 μg/mL or 250 μg/mL DM extract and IRT decreased viability from 62% (treated with IRT alone) to 42% and 30%, respectively (Figure 2C,D).

To check whether sequential treatment could improve the response, cells were pretreated for 3 h with 100 or 250 μg/mL DM extract before being exposed to IRT (100 µM) for 24 h after removing the extract. The pretreatment regimen did not confer any additional cytotoxic benefit over IRT alone on any of the cell lines examined.

### 3.3. Selective Cytotoxic Activity of J. rubens DM Extract in Colon Cancer Cells

**Figure 3:**
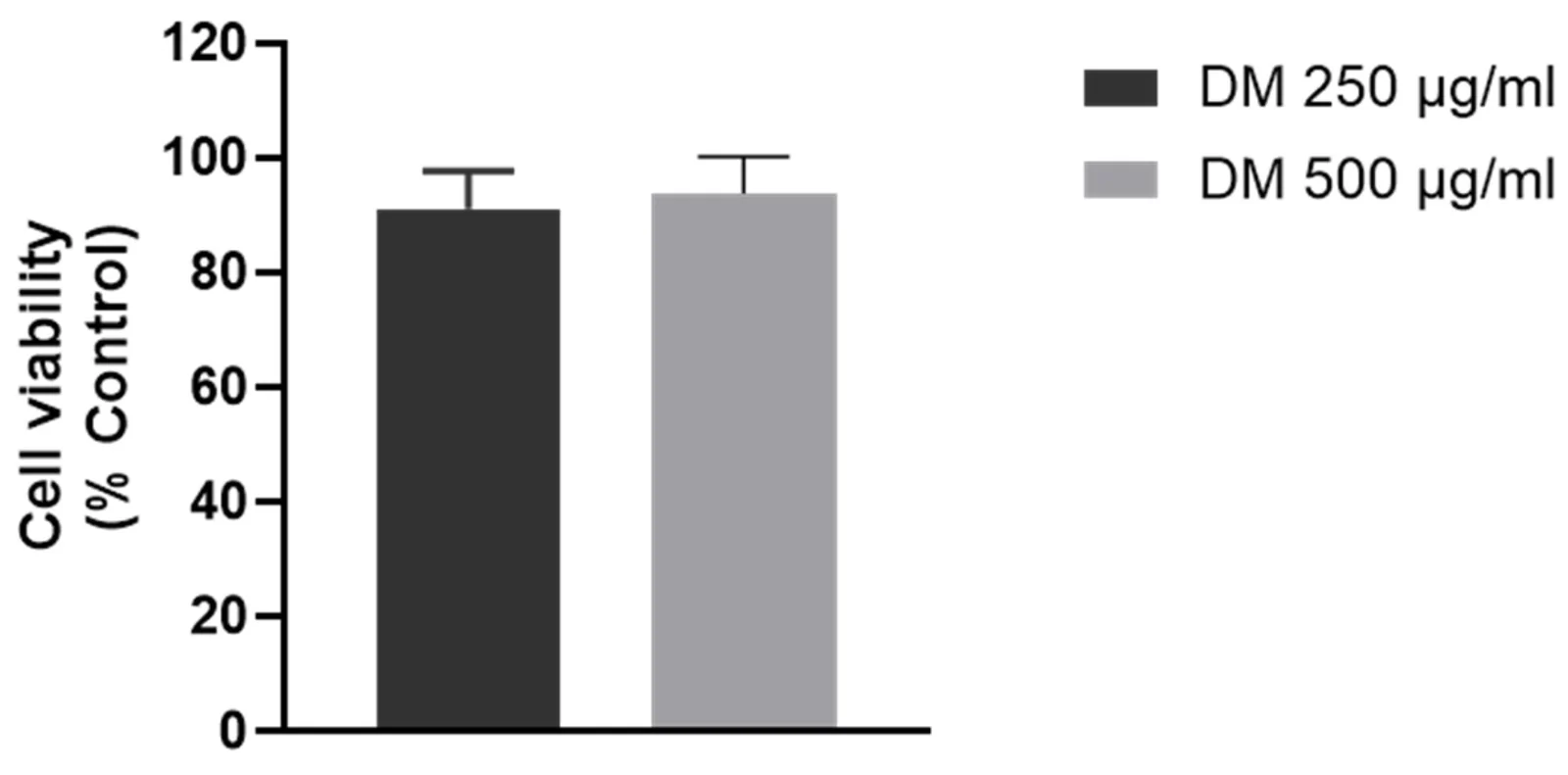
The effect of DM extract on normal-like human cells. MTT assay measured the viability of NCM460D cells. NCM460D cells were treated with DM extract (250 and 500 µg/mL). Cell viability was calculated relative to the control vehicle, which was set to 100%. Results are presented as the mean ± SD (n = 3 independent experiments).

The cytotoxic effect of the DM extract on the non-tumorigenic human colon epithelial cell line NCM460D was evaluated using the MTT assay (Figure 3). Treatment with the extract resulted in only a minimal reduction in cell viability, with NCM460D cells maintaining high viability (>90%), specifically 91% and 94% at concentrations of 250 µg/mL and 500 µg/mL, respectively. These findings indicate a limited cytotoxic effect of the extract on normal-like colon cells and no evident dose-dependent toxicity under the tested conditions, suggesting a degree of selectivity toward colorectal cancer cells.

### 3.4. The DM Soxhlet Extract Potentiates CAP and IRT to Suppress Long-Term Survival of Colorectal Cancer Cells

Based on their reduced sensitivity, HT-29 cells were not included in subsequent assays, and further experiments focused on the more responsive HCT-116 and Caco-2 cell lines.

In order to evaluate the long-term survival and self-renewal capacity of HCT-116 and Caco-2 cancer cells after combination treatment, the cells were treated for 24 h and subsequently allowed to proliferate for one week.

**Figure 4:**
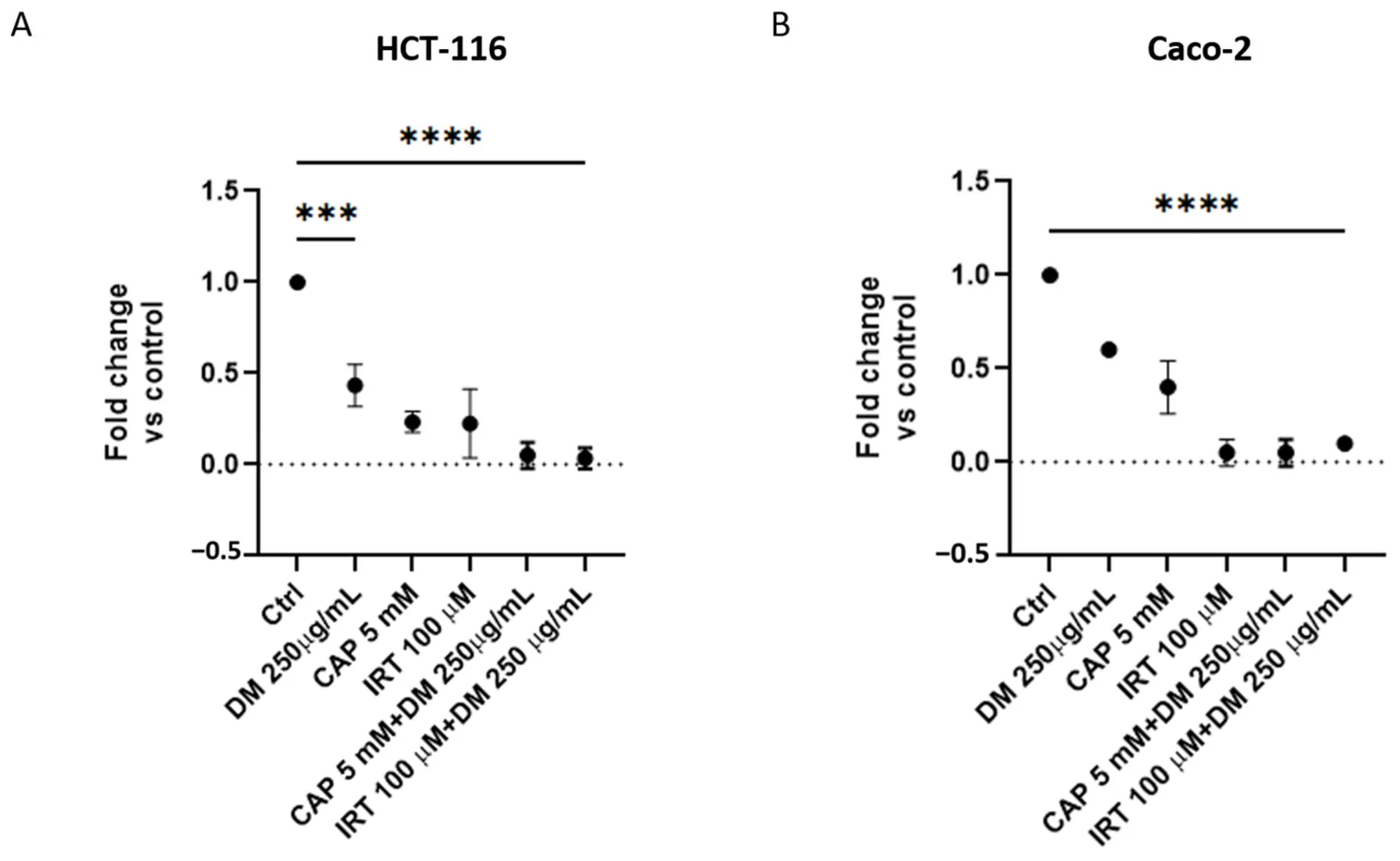
Effect of CAP–DM and IRT–DM combinations on colony formation in HCT-116 and Caco-2 cells. HCT-116 (**A**) and Caco-2 (**B**) colorectal cancer cells were treated with the DM Soxhlet extract (250 µg/mL), capecitabine (CAP, 5 mM), or irinotecan (IRT, 100 µM), individually or in combination for 24 h. The remaining cell colonies were quantified using a crystal violet assay, and results were expressed as fold change relative to the control (set to 1.0). The dotted horizontal line indicates the zero reference (baseline) on the y-axis. Data are presented as mean ± SD (n = 2–4 independent experiments). DM: dichloromethane/methanol extract; CAP: capecitabine; IRT: irinotecan; Ctrl: control. *** *p* < 0.001, **** *p* < 0.0001 vs. Ctrl.

The crystal violet staining demonstrated an apparent reduction in the number of adherent cells following treatment with the DM Soxhlet extract, chemotherapeutic drugs, or their combinations. The DM extract (250 µg/mL) alone reduced the number to ~0.5 (~50% reduction), CAP (5 mM) to ~0.35 (~65% reduction), and IRT (100 µM) to ~0.20 (~80% reduction) in the HCT-116 cells. Again, co-treatment augmented these effects, with CAP + DM and IRT + DM treatments lowering the number to ~0.05–0.10 (~90–95% reduction), indicating a notably greater inhibition of adherent cell mass than single treatments (Figure 4A).

Similarly, in Caco-2 cells, a single treatment with DM extract (250 µg/mL) caused a moderate reduction (~0.6), while CAP (5 mM) and IRT (100 µM) treatments lowered the signal to ~0.45 and ~0.05, respectively. The combination treatments showed the most significant effects, with CAP + DM approaching baseline values (~0–0.05) and IRT + DM also remaining exceptionally low. These results demonstrate that the DM Soxhlet extract enhances the cytotoxic and anti-adhesive action of both CAP and IRT, with highly significant potentiation in co-treated cells (Figure 4B).

### 3.5. DM Soxhlet Extract Selectively Enhances the Anti-Migratory Effect of IRT but Not CAP in HCT-116 Cells

**Figure 5:**
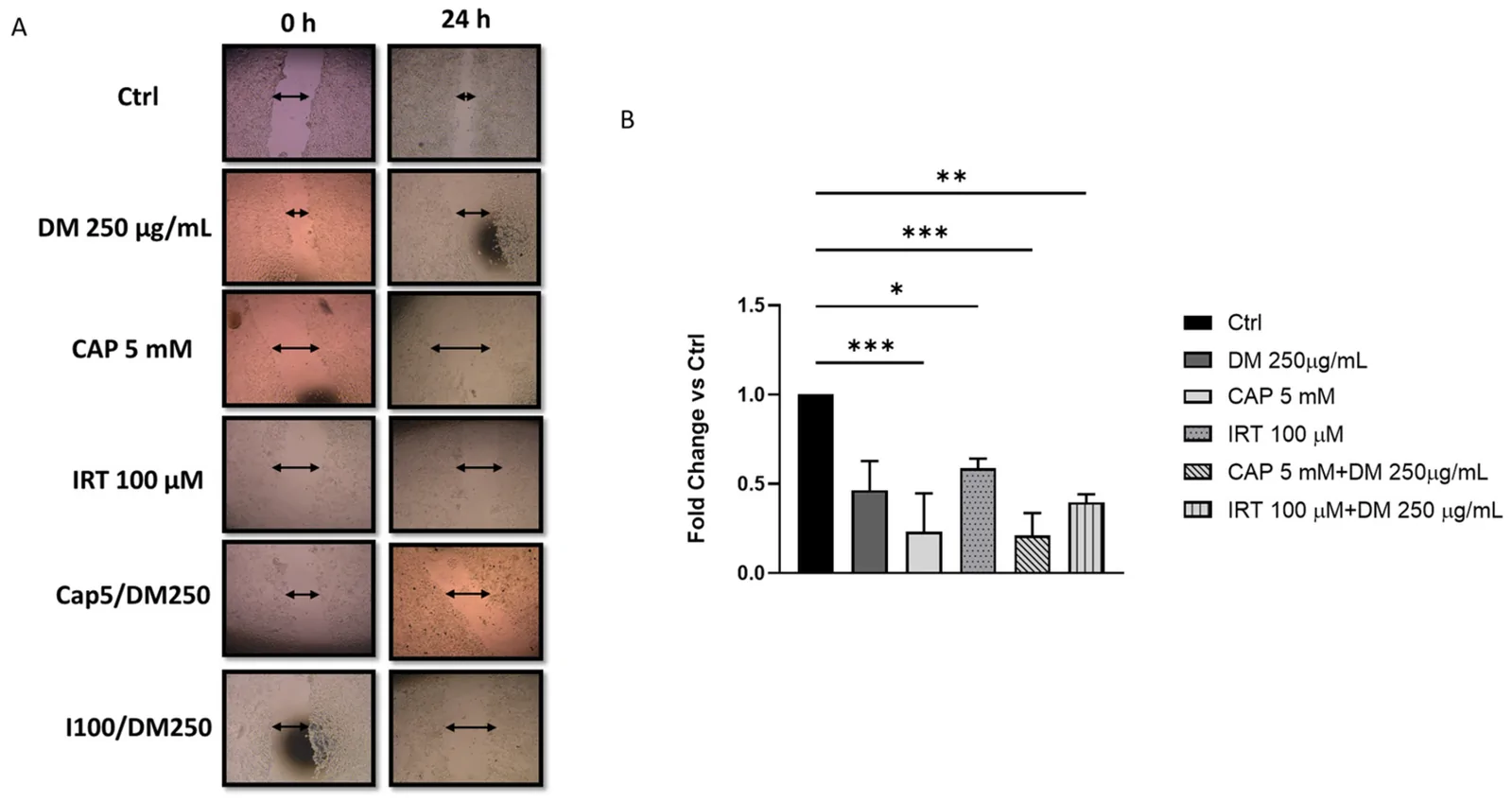
The anti-migratory potential of CAP–DM and IRT–DM combinations in HCT-116 cells. HCT-116 cells were treated with the DM Soxhlet extract (250 µg/mL) and either capecitabine (CAP, 5 mM) or irinotecan (IRT, 100 µM), individually or in combination. (**A**) Representative images of scratched HCT-116 monolayers captured at 0 h and 24 h using a Leica microscope at 4× magnification. The double-headed arrows indicate the remaining wound gap at the indicated time points. (**B**) Bar graphs represent the relative fold change in wound closure after 24 h compared to the control, which was set to 1. Data are presented as mean ± SD (n = 3 independent experiments). DM: dichloromethane/methanol extract; CAP: capecitabine; IRT: irinotecan; Ctrl: control. * *p* < 0.05, ** *p* < 0.01, *** *p* < 0.001 vs. Ctrl.

Since co-treatment with CAP and the DM Soxhlet extract was shown in the MTT assay to significantly inhibit cell growth, a wound-healing assay was subsequently performed to examine their anti- migratory activity in HCT-116. The CAP (5 mM) treatment alone suppressed wound closure to 0.5 relative to the control, and its co-treatment with the DM Soxhlet extract (250 µg/mL) did not exhibit any further inhibitory effect, indicating no impact on cell migration. In comparison, IRT (100 µM) alone suppressed wound closure to 0.6, and its combination with the DM extract further suppressed motility, decreasing the closure ratio to 0.4. These findings indicate that the DM Soxhlet extract selectively potentiates the anti-migratory effect of IRT but not CAP in HCT-116 cells (Figure 5).

### 3.6. DM Soxhlet Extract Potentiates the Anti-Migratory Effects of CAP and IRT in Caco-2 Cells

**Figure 6:**
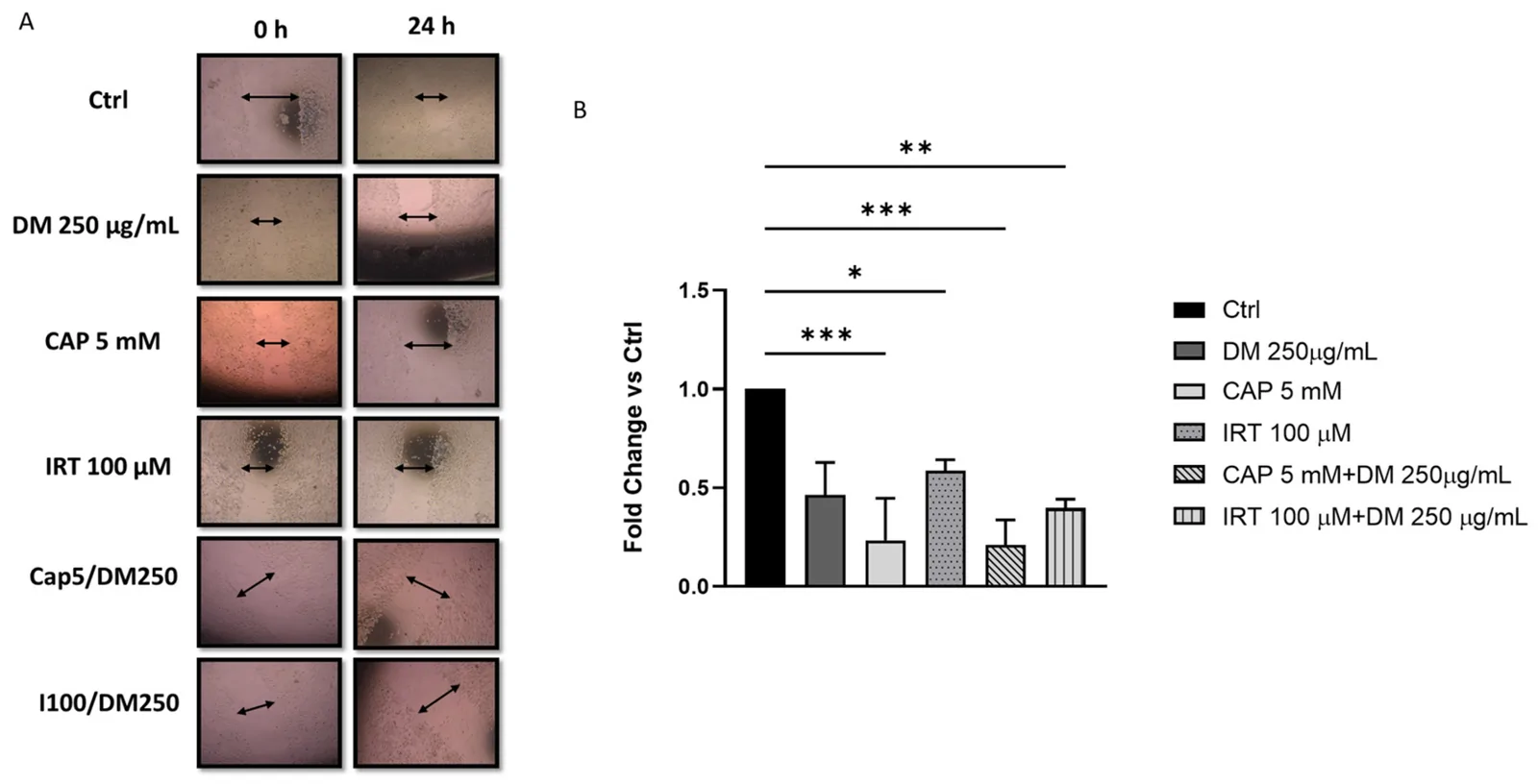
The anti-migratory potential of CAP–DM and IRT–DM combinations in Caco-2 cells. Caco-2 cells were treated with the DM Soxhlet extract (250 µg/mL) in combination with either capecitabine (CAP, 5 mM) or irinotecan (IRT, 100 µM), as well as with each agent individually. (**A**) Representative images of scratched Caco-2 monolayers captured at 0 h and 24 h using a Leica microscope at 4× magnification. The double-headed arrows indicate the remaining wound gap at the indicated time points. (**B**) Bar graphs depicting the relative fold change in wound closure after 24 h compared to the control which was set to 1. Data are expressed as mean ± SD (n = 3 independent experiments). DM: dichloromethane/methanol extract; CAP: capecitabine; IRT: irinotecan; Ctrl: control. * *p* < 0.05, ** *p* < 0.01, *** *p* < 0.001 vs. Ctrl.

In Caco-2 cells, the DM Soxhlet extract amplified the migration-inhibitory effect of both CAP and IRT. CAP (5 mM) alone reduced wound closure to 0.8 compared to the control, and in combination with the DM extract (250 µg/mL), wound closure was further reduced to 0.5. Likewise, IRT (100 µM) alone reduced wound closure to 0.6-fold, and when combined with the DM extract, wound closure was decreased to 0.3. These results show that the DM Soxhlet extract effectively augments the anti-migratory activity of traditional chemotherapeutic drugs in HCT-116 and Caco-2 cells (Figure 6).

### 3.7. DM Extract Promotes Apoptotic Cell Death in a Dose-Dependent Manner in HCT-116 Cells

Given the observed suppression of key pro-survival signaling pathways, we next examined whether DM treatment directly induces apoptotic cell death in HCT-116 cells. Therefore, flow cytometric analysis using Annexin V-FITC/PI staining was performed to evaluate the effect of DM extract (250 and 500 µg/mL) on HCT-116 cell death distribution compared with untreated control cells.

**Figure 7:**
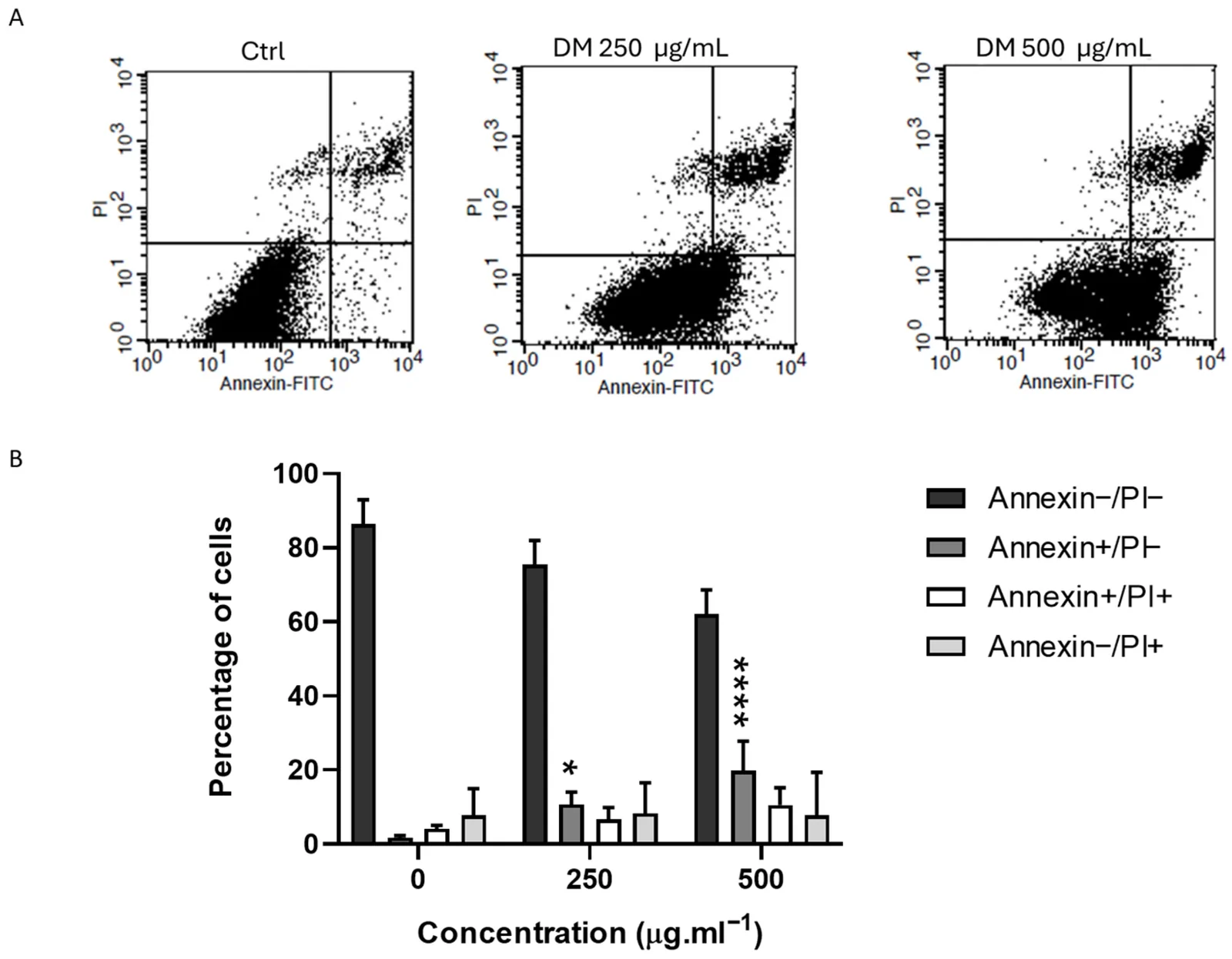
DM extract induces dose-dependent apoptosis in HCT-116 cells. HCT-116 cells were treated with DM extract (250 and 500 µg/mL) or left untreated (Ctrl). (**A**) Representative Annexin V-FITC/PI flow cytometry dot plots show the distribution of viable (Annexin V^−^/PI^−^), early apoptotic (Annexin V^+^/PI^−^), late apoptotic (Annexin V^+^/PI^+^), and necrotic (Annexin V^−^/PI^+^) cell populations. (**B**) Quantitative analysis of the different cell populations following treatment. Data are presented as mean ± SD (n = 3 independent experiments, each performed in duplicate). * *p* < 0.05, **** *p* < 0.0001 versus control.

In the control group, the majority of cells were located in the lower left (LL) quadrant (Annexin−/PI−; 90%), indicating a predominantly viable population with minimal apoptotic cells. Treatment with DM at 250 µg/mL resulted in a reduction in viable cells accompanied by an increase in apoptotic populations. Specifically, the percentage of early apoptotic cells (Annexin+/PI−; LR quadrant) increased to approximately 10%, compared to 2% in the control group. A more pronounced effect was observed following treatment with DM at 500 µg/mL, where a marked increase in both early and late apoptotic populations was detected, reaching approximately 20% and 10%, respectively (Figure 7).

Overall, these findings demonstrate a dose-dependent induction of apoptosis by the DM extract in HCT-116 cells.

### 3.8. DM Extract Downregulates BCL-2 Protein Expression in HCT-116 Cells

**Figure 8:**
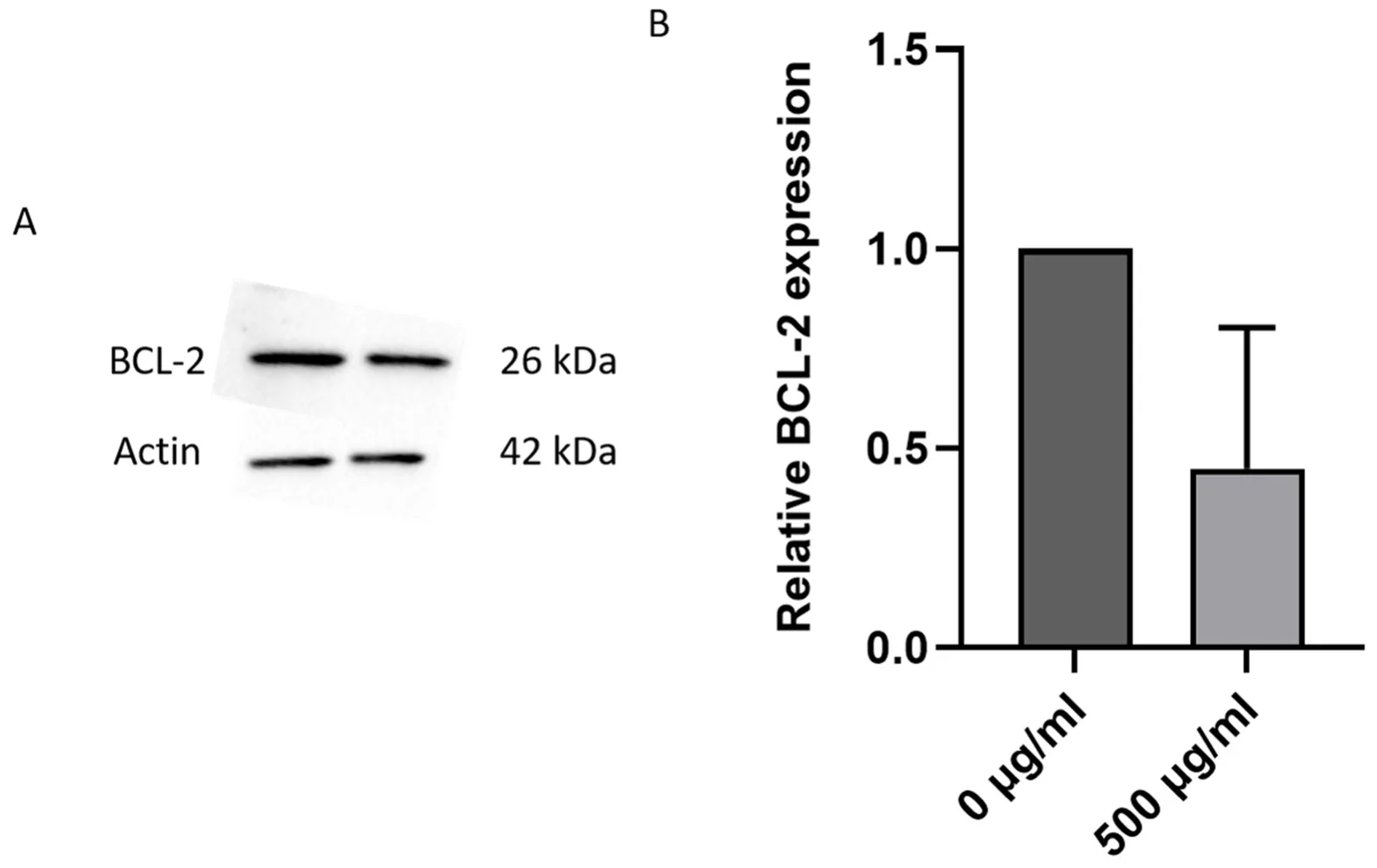
DM extract reduces BCL-2 protein expression in HCT-116 cells. (**A**) Representative Western blot showing BCL-2 and β-actin protein expression in untreated control and HCT-116 cells treated with 500 μg/mL *J. rubens* dichloromethane–methanol (DM) extract for 24 h. (**B**) Densitometric analysis of BCL-2 protein expression normalized to β-actin and expressed as fold change relative to the untreated control. Data represent two independent experiments (n = 2).

To gain additional insight into the molecular pathway of apoptosis induced by DM, the BCL-2 protein was measured in HCT-116 cells by Western blot analysis. As shown in Figure 8, the representative Western blots reveal a decrease in BCL-2 protein levels upon treatment of cells with 500 μg/mL DM extract relative to the untreated control group. The densitometry of normalized bands revealed average BCL-2 expression levels of 0.5-fold relative to the control (Figure 8). These findings are consistent with the Annexin V-FITC/PI results and suggest that downregulation of BCL-2 contributes to the pro-apoptotic activity of the DM extract.

### 3.9. Jania rubens DM Extract Upregulated Pro-Inflammatory Cytokines in HCT-116 Cells

**Figure 9:**
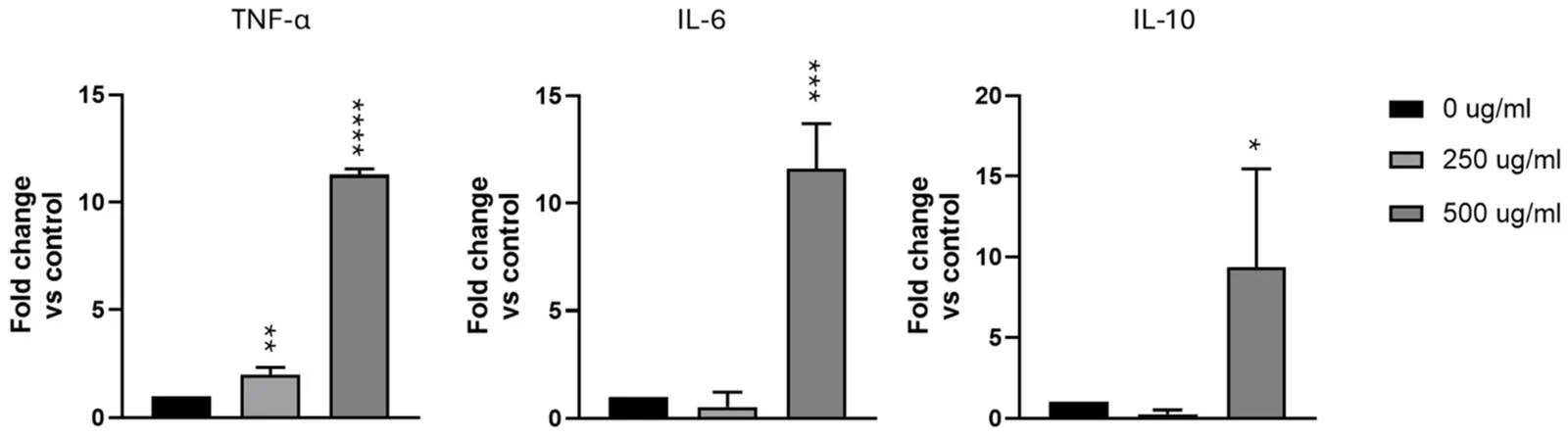
The effect of *J. rubens* DM extract on pro-inflammatory cytokines in HCT-116 cells. Relative mRNA expression levels of TNF-α, IL-6, and IL-10 were quantified. HCT-116 cells were either left untreated or treated with 250 or 500 μg/mL DM extract for 16 h. QRT-PCR quantified gene expression, normalized to GAPDH and presented relative to untreated control cells. Results are presented as the mean ± SD from three independent experiments. * *p* < 0.05, ** *p* < 0.01,*** *p* < 0.001, **** *p* < 0.0001.

Given the role of inflammatory mediators in regulating tumor cell survival and chemotherapeutic responsiveness, we evaluated whether DM treatment alters the expression of key cytokines in HCT-116 cells. RT-PCR results showed that the mRNA expression of TNF-α significantly increased in response to DM treatment at concentrations of 250 and 500 μg/mL. However, transcriptional expressions of IL-6 and IL-10 significantly increased only at the higher DM concentration (500 μg/mL) (Figure 9). These findings indicate that DM extract modulates tumor cell-derived cytokine expression, suggesting a potential involvement of cytokine-related signaling pathways in its anticancer effects.

### 3.10. Jania rubens DM Extract Potentiates Irinotecan-Induced Apoptosis via Modulation of BCL-2 Expression in HCT-116 Cells

**Figure 10:**
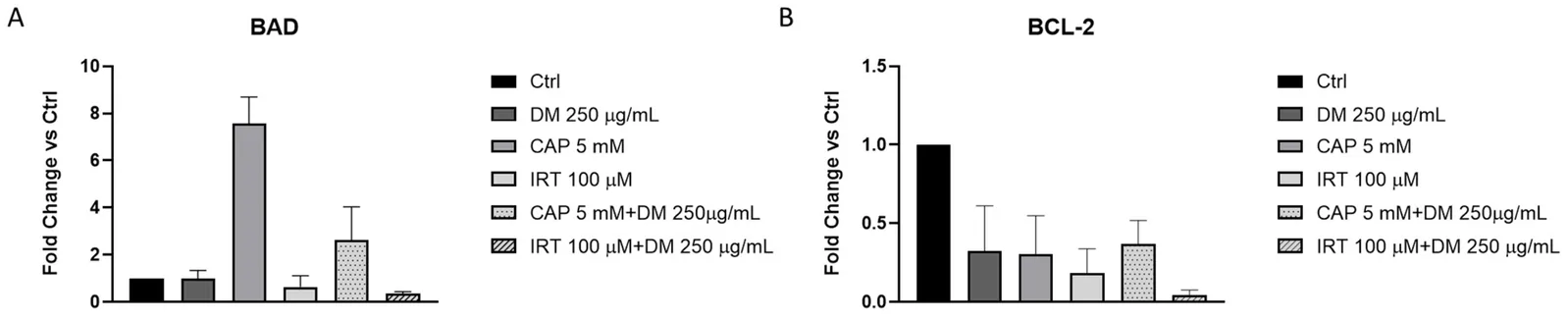
The effect of CAP–DM and IRT–DM Combinations on BAD and BCL-2 m-RNA levels. (**A**) The relative mRNA expression of BAD was evaluated in HCT-116 cells following 16 h treatment with the DM Soxhlet extract (250 µg/mL) in combination with either capecitabine (CAP, 5 mM) or irinotecan (IRT, 100 µM), as well as with each agent individually. (**B**) Relative BCL-2 mRNA expression in HCT-116 cells under the same treatment conditions. Transcript levels were determined by qRT-PCR, normalized to GAPDH, and expressed as fold change relative to untreated controls. Data are shown as mean ± SD from three independent experiments.

Given the pro-apoptotic effect of DM treatment, we further investigated whether co-administration with CAP and IRI enhances apoptotic induction and whether this effect is associated with alterations in apoptotic signaling. HCT-116 cells were treated for 16 h with the DM Soxhlet extract (250 µg/mL), either alone or in combination with capecitabine (CAP, 5 mM) or irinotecan (IRT, 100 µM), as well as with each agent individually. Quantitative RT-PCR demonstrated a robust modulation of apoptotic gene expression in HCT-116 colorectal cancer cells in response to the different treatment regimens. Findings indicated differences in BAD and BCL-2 mRNA levels in response to various treatments. As shown in Figure 8, BAD expression was considerably elevated in capecitabine-treated cells (7.55 ± 1.14-fold vs. control, *p* < 0.001), showing strong pro-apoptotic induction. Co-treatment with the *J. rubens* DM extract reduced this induction (2.63 ± 1.40-fold), suggesting that DM somewhat inhibits CAP-induced mitochondrial stress (Figure 10A).

On the other hand, BCL-2 was highly downregulated in most treatment groups compared to control (1.00 ± 0.00), with the strongest downregulation observed in the IRI 100 µM + DM 250 µg/mL combination group (0.04 ± 0.03) (*p* < 0.05), reflecting the inhibition of anti-apoptotic signals. Notably, BCL-2 expression was also reduced by IRI 100 µM alone (0.18 ± 0.15), but the addition of DM further enhanced this effect. Overall, co-administration of the DM extract with IRT increased apoptotic signaling by concurrently suppressing anti-apoptotic BCL-2, while capecitabine alone primarily enhanced BAD expression. These data indicate that the DM extract promotes IRT activity most effectively by inducing apoptosis in HCT-116 cells through mitochondria-mediated mechanisms (Figure 10B).

## 4. Discussion

Chemoresistance and treatment-induced toxicity are still significant issues in the treatment of colorectal cancer (CRC), thereby reducing the long-term effectiveness of traditional chemotherapeutic drugs such as CAP and IRT [7,10]. The persistent activation of pro-survival pathways and resistance to apoptosis are key contributing factors to chemoresistance and recurrence [26,27]. In this scenario, multi-target approaches that can modulate multiple pathways associated with resistance have attracted increasing attention [28]. Natural compounds, especially pharmacological agents, have emerged as a promising strategy for targeting multiple molecular mechanisms rather than a single pathway [29]. In the current study, we show that the dichloromethane–methanol (DM) extract of *J. rubens* can potentiate the antitumor activity of CAP and IRT by suppressing survival signaling, regulating apoptotic modulators, and altering inflammatory mediators.

Our findings demonstrate that the *J. rubens* DM extract exerts differential effects in CRC cell lines when combined with CAP or IRT. In HCT-116 and Caco-2 cells, co-treatment significantly enhanced drug-induced cytotoxicity, whereas pretreatment did not alter treatment response. This simultaneous exposure of cancer cells to the DM extract and chemotherapeutic agents could potentiate drug-induced cellular damage during the early phase of the treatment. This could be initiated through the modulation of redox balance and stress-associated signaling pathways.

Importantly, while enhanced effects were observed during co-treatment, the absence of a consistent response in the pretreatment setting suggests that these interactions should be interpreted with caution. Without formal pharmacological evaluation of these interactions, the effects are better characterized as additive. However, since potentiation was consistently observed across different models, this interaction has biological significance and should be studied through proper combination studies.

Mechanistically, these findings are consistent with our previous observations that the DM extract alone induces cell-cycle arrest and suppresses EMT and metastasis-related markers in HCT-116 cells through ROS elevation and TET downregulation [24]. Increased ROS levels can disrupt mitochondrial function, impair DNA repair processes, and sensitize cells to agents that target nucleic acid integrity [30]. CAP, through its active metabolite 5-FU, interferes with thymidylate synthase-dependent dTMP synthesis and RNA processing [31], whereas IRT stabilizes topoisomerase I–DNA cleavage complexes [32]. In this context, a ROS-imbalanced intracellular environment, potentially accompanied by epigenetic alterations such as reduced 5-hmC levels, may facilitate apoptosis induction [24]. Consistently, DM treatment also reduced migratory capacity in wound-healing assays, correlating with EMT repression and loss of motility.

We have also shown that the DM Soxhlet extract has a complex mixture of bioactive compounds, mainly flavonoid derivatives [24]. Flavonoids have been extensively documented to increase the potency of standard chemotherapeutic agents by modulating oxidative stress, apoptosis, and survival pathways. For instance, quercetin was found to enhance CAP-induced cytotoxicity by inducing mitochondrial damage and caspase activation [33]. In contrast, silymarin was found to improve the effectiveness of CAP or IRT in colon cancer models by activating pro-apoptotic pathways and downregulating BCL-2 expression [34]. These observations suggest that flavonoid extracts can serve as useful chemosensitizers by modulating multiple pathways.

The present study demonstrated that the DM Soxhlet extract enhanced the cytotoxicity of CAP and IRT in HCT-116 and Caco-2 cells but not in HT-29 cells. This response is probably due to the molecular heterogeneity of colorectal carcinomas and emphasizes the role of genetic background in the design of combination therapy. HT-29 cells harbor intrinsic resistance features that may limit responsiveness to ROS-dependent sensitization. Notably, antioxidant defenses in HT-29 cells are regulated by NRF2, which induces protective enzymes such as sulfiredoxin (SRX) [35], potentially counteracting ROS-mediated cytotoxicity. In addition, the presence of mutant p53 in HT-29 cells may impair apoptosis induction following DNA damage or oxidative stress, thereby reducing sensitivity to the combination treatment [36,37,38].

Notably, the combination treatment resulted in a significant decrease in long-term clonogenic survival, suggesting a compromised ability to form colonies following co-administration of the DM extract and chemotherapeutic agents as compared with chemotherapeutic agents used alone. This could emphasize the key role of DM as an adjuvant that could prevent relapse and metastasis by acting on the apoptosis-resistant cells. This result is especially relevant, as traditional chemotherapy has been shown to spare populations of cells with greater proliferative ability, contributing to relapse and metastasis [39]. The DM extract could then be considered as a complement to traditional chemotherapy to inhibit more resistant tumor cell populations.

In HCT-116 cells, RT-qPCR revealed marked changes in the expression of apoptotic regulators following various treatment regimens, suggesting the potential of the DM extract to modulate cell fate in a combination therapy regimen. CAP monotherapy induced a substantial increase in the pro-apoptotic gene BAD, consistent with activation of the intrinsic mitochondrial apoptotic pathway. Although CAP mainly exerts its cytotoxic action by inhibiting DNA and RNA synthesis as an antimetabolite [40], the induction of BAD expression could be a secondary mitochondrial stress response in HCT-116 cells. Co-treatment with the DM extract resulted in a lower induction of BAD expression compared to CAP alone. However, despite this difference, the combination treatment exhibited greater overall cytotoxic and anti-migratory effects in both HCT-116 and Caco-2 cells. Importantly, apoptotic regulation is governed by the balance between multiple pro- and anti-apoptotic factors, and the observed decrease in BAD expression relative to CAP alone does not preclude enhanced apoptosis when concurrent modulation of anti-apoptotic pathways is considered. This suggests that the effect of the DM extract is not solely dependent on BAD transcription, but rather involves additional mechanisms such as increased oxidative stress, inhibition of EMT progression, and impairment of long-term clonogenic survival [24].

In contrast, BCL-2 mRNA expression was markedly reduced across several treatment groups, with the strongest repression observed in the IRT-DM combination. Although IRT alone decreased BCL-2 levels, co-administration with DM further intensified this effect, supporting enhanced apoptosis through inhibition of anti-apoptotic signaling. These findings are consistent with reports that IRT downregulates BCL-2 in HCT-116 cells, promoting mitochondrial permeability and apoptosis [41]. The previously demonstrated capacity of the DM extract to increase ROS and modulate TET-dependent epigenetic pathways may further contribute to this pro-apoptotic shift [24]. Taken together, these findings suggest that the DM extract can potentiate the effects of CAP and IRT by modulating distinct apoptotic regulators. Instead of targeting a single pathway, the DM appears to integrate downregulation of anti-apoptotic pathways and activation of mitochondrial stress responses, thereby confirming its role as a multi-targeted redox modulator in colorectal cancer.

The pro-apoptotic shift detected at the transcriptional level was functionally validated by Annexin V/PI flow cytometry, which revealed a dose-dependent increase in the proportion of apoptotic cells after DM treatment. It is significant to note that the increase in early- and late-apoptotic cell proportions was accompanied by little necrosis, suggesting that DM induces apoptosis rather than nonspecific cytotoxicity. These findings suggest that the DM extract modulates apoptotic signaling in CRC cells, thereby enhancing their response to chemotherapeutic stress.

The observed reduction in BCL-2 protein expression following DM treatment is consistent with the enhanced apoptosis detected by Annexin V-FITC/PI staining and complements the changes in BCL-2 gene expression observed following combination treatment with CAP or IRT. Together, these findings suggest that modulation of the BCL-2 apoptotic pathway contributes to the ability of the DM extract to enhance the response of colorectal cancer cells to chemotherapy.

In addition to its cytotoxic properties, DM treatment also altered the expression of inflammatory cytokines TNF-α, IL-6, and IL-10 in HCT-116 cells. Tumor cell-derived cytokines can participate in autocrine and paracrine signaling circuits that modulate survival signaling, stress responses, and apoptotic sensitivity [42,43]. Both IL-6 and TNF-α have been shown to modulate intracellular signaling pathways such as PI3K/AKT and MAPK/ERK, and their actions are context- and time-dependent, either as pro-survival or pro-apoptotic signals, depending on the tumor microenvironment, including stromal cells and tumor cellular context and signaling intensity [44,45,46,47]. Although IL-6 is a pro-inflammatory cytokine, its role in cancer is highly context- and time-dependent. At early stages, IL-6–mediated inflammation may contribute to the activation of anti-tumor immune responses, whereas at later stages, particularly within the tumor microenvironment (TME), IL-6 produced by senescent or infiltrating cells can promote tumor progression and suppress anti-tumor immunity [48]. In this context, the concurrent induction of IL-10 expression suggests a complex interplay between cytokines, potentially reflecting a regulatory feedback mechanism aimed at resolving the inflammatory response. IL-10 may be induced as part of this regulatory network, possibly downstream of IL-6 signaling; however, further investigation at extended time points is required to determine whether its upregulation reflects a sequential relationship or involves additional signaling mechanisms. The simultaneous induction of IL-10 expression also points to a complex inflammatory response that may reflect adaptive stress-related signaling within cancer cells. These findings suggest that DM extract modulates apoptotic signaling pathways in CRC cells, thereby enhancing cellular responses to chemotherapeutic stress.

Taken together, these results collectively point to the *J. rubens* DM extract as a promising multi-targeted adjuvant that could potentially improve cytotoxic and anti-migratory activities in colorectal cancer cells by modulating survival signaling and apoptotic regulators. Based on its previously established ability to modulate redox regulation and TET-mediated epigenetic modulation, the DM extract could improve chemotherapeutic activity by suppressing survival signaling and apoptotic resistance pathways. Overall, these findings support the potential biological activity of the DM extract and warrant further investigation to evaluate its safety and therapeutic relevance.

## Data Availability

The original contributions presented in this study are included in the article. Further inquiries can be directed to the corresponding authors.

## Abbreviations

The following abbreviations are used in this manuscript:

CRC	Colorectal cancer
DM	Dichloromethane-methanol
CAP	Capecitabine
IRT	Irinotecan
*J. rubens*	*Jania rubens*
ROS	Reactive oxygen species
EMT	Epithelial–mesenchymal transition
